# ‘Off-the-Shelf’ Immunotherapy: Manufacture of CD8^+^ T Cells Derived from Hematopoietic Stem Cells

**DOI:** 10.3390/cells10102631

**Published:** 2021-10-02

**Authors:** Nicholas Boyd, Kellie Cartledge, Huimin Cao, Vera Evtimov, Aleta Pupovac, Alan Trounson, Richard Boyd

**Affiliations:** 1 Cartherics Pty Ltd., Clayton, VIC 3168, Australia; nicholas.boyd@hudson.org.au (N.B.); kellie.cartledge@hudson.org.au (K.C.); huimin.cao@hudson.org.au (H.C.); vera.evtimov@hudson.org.au (V.E.); aleta.pupovac@hudson.org.au (A.P.); alan.trounson@hudson.org.au (A.T.); 2 Department of Obstetrics and Gynaecology, Monash University, Clayton, VIC 3168, Australia

**Keywords:** HSC, CD8^+^ T cells, differentiation, off-the-shelf immunotherapy

## Abstract

Cellular immunotherapy is revolutionizing cancer treatment. However, autologous transplants are complex, costly, and limited by the number and quality of T cells that can be isolated from and expanded for re-infusion into each patient. This paper demonstrates a stromal support cell-free in vitro method for the differentiation of T cells from umbilical cord blood hematopoietic stem cells (HSCs). For each single HSC cell input, approximately 5 × 10^4^ T cells were created with an initial five days of HSC expansion and subsequent T cell differentiation over 49 days. When the induced in vitro differentiated T cells were activated by cytokines and anti-CD3/CD28 beads, CD8^+^ T cell receptor (TCR) γδ^+^ T cells were preferentially generated and elicited cytotoxic function against ovarian cancer cells in vitro. This process of inducing de novo functional T cells offers a possible strategy to increase T cell yields, simplify manufacturing, and reduce costs with application potential for conversion into chimeric antigen receptor (CAR)-T cells for cancer immunotherapy and for allogeneic transplantation to restore immune competence.

## 1. Introduction

Immunotherapy is now a recognized pillar of cancer treatment alongside chemotherapy, radiation, surgery, and therapeutic small molecules. This success is mainly attributed to checkpoint inhibitors and cell therapies such as chimeric antigen receptor (CAR)-T cells [1]. The combination of cancer cell recognition and “supercharged” cytotoxic T cell function has enabled CAR-T cells to realize unprecedented success against certain blood cancers, effectively revolutionizing this field [2,3,4]. Despite the global optimism for CAR-T cells, there are still several fundamental problems associated with current autologous therapies: the age and/or quality of T cells that can be obtained from the donor, the finite number of T cells that can be generated for therapy and a risk of cytokine release syndrome after infusion into the patient [5,6]. Furthermore, the clinical implications related to more generalized T cell deficiencies are wide reaching. For example, there is a clear correlation between immunodeficiency, thymic atrophy in adults and reduced numbers of naive T cells [7,8,9]. This not only leads to poor immunity in the aged, but also has direct consequences on the ability of cancer patients to recover immune competency following myeloablative chemotherapy and rescue hematopoietic stem cell (HSC) transplantation. In particular, the failure to regenerate sufficient naive T cells is a direct causative link to high-risk opportunistic infection and associated morbidity and often mortality [7,10,11]. Allogeneic T cell transplants can offer a solution to this, but may cause graft-versus-host disease (GVHD) [6]. Thus, the utilization of γδ T and natural killer (NK) cells in an allogeneic setting is rapidly growing due to their innate functional characteristics and better safety profiles [12,13].

One logical approach to overcoming T cell-based immunodeficiency would be to derive lymphocytes ex vivo from appropriate stem cell sources. We, and others, are currently using human induced pluripotent stem cells (iPSCs) as a theoretically limitless resource for inducing T cells and NK cells. However, this has mainly been achieved with the use of murine stromal support lines [14,15,16,17,18,19,20,21,22,23,24], which are difficult to implement clinically and may be of concern for regulatory approval. An alternative approach could be to use umbilical cord blood (UCB) as an enriched source of HSCs, which are the ultimate in vivo progenitors of T cells [25]. Moreover, vast numbers of cord samples have been cryopreserved globally in both public and private banks, theoretically providing a huge resource of potential donors. However, while cord blood is HSC enriched, there is still a limited absolute number that can be obtained from any one donor. Recent studies have indicated that HSCs can be induced to self-renew ex vivo to some degree [26,27,28,29], which may help address this.

The present study provides a fully defined approach to induce T cells from cord HSCs without any need for co-culture stromal support lines, providing advances in manufacture simplicity, scope for scalability and circumventing potential regulatory issues. Despite evident hurdles for clinical translation, this method serves to address at least one aspect of the unmet clinical need for ‘off-the-shelf’ anti-cancer immunotherapies. It also provides a possible option to replenish the T cell-based immune system in more generalized immune deficiency states linked to myeloablative cancer chemotherapy, prolonged infection, and the immune cell needs of the ever-increasingly aged population.

## 2. Materials and Methods

### 2.1. CD34^+^ Cell Preparation and Expansion from UCBs

UCBs were obtained from full-term elective caesarean section volunteers from the Murdoch Children’s Research Institute of Royal Children’s Hospital, under Material Transfer agreement #MTA 24131. UCB samples were stored at room temperature and processed within 48 h of collection. Cord blood mononuclear cells (CBMCs) were isolated by Ficoll^™^ Paque (GE Healthcare, Uppsala, Sweden) gradient centrifugation and CD34^+^ cells were enriched using the Cell Depletion MicroBead Kit followed by the CD34^+^ MicroBead Kit (Miltenyi Biotec. Inc., Bergisch Gladbach, Germany). The cell number and purity of the enriched CD34^+^ fraction was analyzed by the TC20 cell counter (Bio-Rad, Hercules, CA, USA) with trypan blue staining and the MACSQuant^®^ flow cytometer (Miltenyi Biotec. Inc., Bergisch Gladbach, Germany). The purity obtained was greater than 90%. CD34^+^ cells were seeded at a density of 1 × 10^5^ cells/mL and cultured at 37 °C, 5% CO_2_, in CD34 Expansion media consisting of StemSpan^™^ SFEM II (STEMCELL Technologies, Vancouver, BC, Canada) supplemented with a human cytokine cocktail of 100 ng/mL recombinant stem cell factor (SCF), 100 ng/mL recombinant thrombopoietin (TPO), 100 ng/mL recombinant Fms-related tyrosine kinase 3 ligand (Flt-3L) and 50 ng/mL recombinant interleukin-6 (IL-6) (all Miltenyi Biotec. Inc., Bergisch Gladbach, Germany) for 5 days. These expanded CD34^+^ cells were cryopreserved in CryoStor^®^ (Sigma-Aldrich, St. Louis, MI, USA).

### 2.2. T Cell Differentiation Assay

Cryopreserved CD34^+^ cells previously expanded for 5 days, were allowed to recover after thawing by 1 day of culture at a density of 3–4 × 10^5^ cells/mL in StemSpan^™^ II (STEMCELL Technologies, Vancouver, BC, Canada) supplemented with a human cytokine cocktail consisting of 100 ng/mL SCF, 100 ng/mL TPO, 100 ng/mL Flt-3L and 50 ng/mL IL-6. CD34^+^ UCB cells were then adjusted to 2.5 × 10^3^ cells/cm^2^ into 6 cm tissue culture plates pre-coated with StemSpan^™^ Lymphoid Differentiation coating material (STEMCELL Technologies, Vancouver, BC, Canada) in media consisting of StemSpan^™^ II supplemented with StemSpan^™^ Lymphoid Progenitor Expansion Supplement (10X) (STEMCELL Technologies, Vancouver, BC, Canada). This timepoint was denoted Day 0 of differentiation. During the first 14 days, cell cultures were refreshed with new media every 3–4 days. At Day 7 and 14 respectively, cell counts and viability assessments were performed using the TC20 cell counter via trypan blue staining. At Day 14 these cells were then further differentiated in StemSpan^™^ II supplemented with StemSpan^™^ Lymphoid Progenitor Expansion Supplement (10X), IL-7 and Flt-3L (collectively referred to as Mature media). Mature media was refreshed every 3–4 days from Day 14 onwards. For each week of culture, total numbers of differentiated progenitor-T (Pro-T) and T cells were calculated via characterization of each cell subset using flow cytometry (described in Section 2.3), as a proportion of total live cells in culture. Cumulative fold expansion relative to the initial cell seeding number was also calculated according to the equation: fold change = total number of live cells obtained at the end of a given culture period/the total number of live cells seeded at the beginning of the given culture period. At Day 42 of differentiation, immature T cells were re-cultured for a further 7 days at 2 × 10^6^ cells/mL into 6 well tissue culture plates in StemSpan^™^ II supplemented with StemSpan^™^ Lymphoid Progenitor Expansion Supplement (10X) and cytokines as described in Etzensperger et al. [30] (collectively referred to as 6F Media). To induce the final stage of differentiation and functional maturity, the T cells were cultured with anti-CD3/CD28 DynaBeads^®^ (Life Technologies, Carlsbad, CA, USA) at a 1:1 bead to cell ratio in 6F Mature media at a cell density of 0.25–0.5 × 10^6^ cells/mL for the first 3–4 days of the additional 7-day culture. Following this stimulation, DynaBeads^®^ were magnetically removed and a complete media change was performed, placing cells back into 6F Mature media. The resultant differentiated T cells at Day 49 were collected from culture and used in downstream functional assays. Cultures were maintained in a 37 °C, 5% CO_2_ incubator throughout. 

### 2.3. Cell Surface Marker Expression on Differentiated T Cells

Expression of cell surface markers on differentiated T cells was determined using the MACSQuant^®^ flow cytometer. Briefly, cells were harvested from culture at indicated time points and incubated with the appropriate concentration of monoclonal antibody (Table S1) with Tandem Signal Enhancer (Miltenyi Biotec. Inc., Bergisch Gladbach, Germany) in flow cytometry staining buffer (dPBS, 0.5% bovine serum albumin, 0.5 mM EDTA) for 10 min at 4 °C. Cells were washed once by centrifugation, and propidium iodide (Miltenyi Biotec. Inc., Bergisch Gladbach, Germany) was added to exclude dead cells. Data were analyzed using the FlowLogic^™^ software (Miltenyi Biotec. Inc., Bergisch Gladbach, Germany). Staining controls included: unstained cells, peripheral blood mononuclear cells and isotype-matched control antibodies. All antibodies, including isotype controls, were purchased from Miltenyi Biotec. Inc. (Supplementary Table S1).

### 2.4. CBMC-Derived T Cells

CBMCs were isolated by Ficoll^™^ Paque centrifugation using Leucosep^™^ tubes (Greiner, Kremsmunster, Austria) as per manufacturer’s instructions. CBMCs were cryopreserved prior to use. T cells were isolated from freshly thawed CBMCs using anti-CD3/CD28 DynaBeads^®^ as per the manufacturer’s instructions. CBMC T cell cultures were maintained in T cell expansion media comprising of IL-2, IL-7, IL-15, IL-21 (Miltenyi Biotec, Bergisch Gladbach, Germany), human AB serum (hAB; Sigma, St. Louis, MI, USA), and Stemulate^®^ (Cook Regentec, Indianapolis, IN, USA) in TexMACS^™^ (Miltenyi Biotec, Bergisch Gladbach, Germany) for continued expansion.

### 2.5. Cell Lines

All cell lines were acquired from the American Type Culture Collection (Manassas, VA, USA) and maintained using recommended culture conditions. The ovarian cancer cell line OVCAR-3 (HTB-161) was maintained in RPMI-1640 (Sigma-Aldrich, St. Louis, MI, USA) supplemented with 20% fetal bovine serum (FBS, Bovogen, Keilor East, Australia), 0.01mg/mL bovine insulin (Sigma-Aldrich, St. Louis, MI, USA) and 1× penicillin-streptomycin (Gibco, Waltham, MA, USA). The ovarian cancer cell line derived from ascites MES-OV (CRL-3272) was cultured in McCoy’s 5a Medium Modified (Gibco, Waltham, MA, USA) containing 10% FBS (Bovogen, Keilor East, Australia) and 1× penicillin-streptomycin (Gibco, Waltham, MA, USA). Cell lines were maintained at 37 °C, 5% CO_2_.

### 2.6. In Vitro Cytotoxicity Assay

The Real Time Cell Analyzer (RTCA), xCELLigence (ACEA Biosciences, San Diego, CA, USA) was employed to determine the ability for HSC-derived T cells to elicit cytotoxic function in vitro. In brief, xCELLigence utilizes electronic sensor technology to assess the local ionic environment at the interface of an electrode and solution. Cellular adherence and an increase in cell number correlate with an increase in electrode impedance (expressed as the arbitrary unit Cell Index (CI)). Conversely, a reduction in CI is indicative of target cell death [31]. The adherent ovarian cancer cell lines OVCAR-3 and MES-OV respectively were plated at 1 × 10^4^ cells/well of an xCELLigence 96-well sensor plate. Cells were maintained at 37 °C, 5% CO_2_ in cell line-specific media for 4–6 h to allow for cellular attachment. Following establishment of target cell monolayer, effector cells were added at two effector to target ratios (E:T, 5:1 or 1:1). Effector cells used herein were unsorted and included all live cells in the final culture. T cells isolated from CBMCs were functionally analyzed in parallel at comparable E:T ratios. Co-cultures were maintained for at least 20 h. Throughout, CI was monitored at 15 min read intervals. The RTCA software version 2.1.0 was used to normalize data to the time of addition of effector cells (expressed as Normalized CI) using the following equation: Normalized CI_ti_ = CI_ti_/CI_nml_time_. Percent cytotoxicity was calculated using the following equation: ((Av. Normalized CI_control_ − Normalized CI_test_)/Av. Normalized CI_control_) × 100. 

### 2.7. Statistical Analysis

Data represent mean, mean ± standard error of the mean (SEM) or mean ± standard deviation (SD) from at least four biological replicates unless otherwise stated. GraphPad Prism 8.0 was used to perform statistical analysis throughout. Results were analyzed using an unpaired *t*-test for two group comparisons when equal variance was confirmed using the F test. A Welch’s *t*-test was used for two group comparisons with unequal variance. A one-way ANOVA with Tukey’s post hoc analysis was implemented for multi-group comparisons. For cytotoxic function, results were analyzed using a Welch’s *t*-test for two group comparison and a one-way ANOVA with the Brown–Forsythe and Welch test for multi-group analysis. Statistical significance was defined as *p* ≤ 0.05. Significance is denoted on graphs as * *p* ≤ 0.05, ** *p* ≤ 0.01, *** *p* ≤ 0.001 or **** *p* ≤ 0.0001. 

## 3. Results

### 3.1. UCB-Derived CD34^+^ Expansion and Differentiation to T Cells

**Figure 1:**
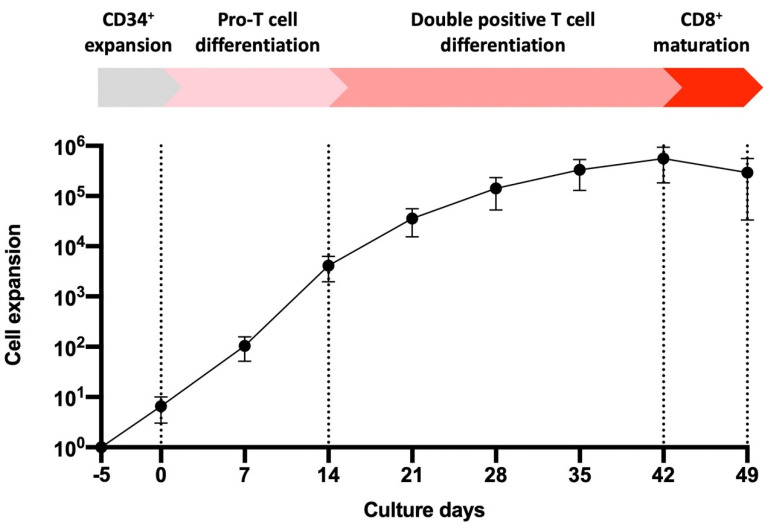
Umbilical cord blood (UCB)-derived CD34^+^ cell expansion and differentiation to T cells. Schematic of the HSC to T cell differentiation method. UCB-derived CD34^+^ cells were isolated and initially expanded for 5 days in CD34 Expansion media (Day −5–Day 0, CD34^+^ expansion step). Differentiation to Pro-T cells was induced over 14 days (Day 0–Day 14, Pro-T cell differentiation step) and Pro-T cells to double positive (DP) T cells over an additional 28 days of differentiation (Day 14–Day 42, Double positive T cell differentiation step) in Mature media. DP to single positive (SP) T cell transition was induced by activation in cytokines for a further 7 days of differentiation (Day 42–Day 49, CD8^+^ maturation step) in 6F Media together with anti-CD3/CD28 bead stimulation for the first 3–4 days (CD8^+^ maturation step). Cumulative fold change of total live cells relative to a single HSC is shown at all steps of T cell differentiation over 49 days of culture. Data points and error bars indicate the mean fold change ± standard deviation (SD) from 5 representative UCB samples. Colors represent differentiation steps as indicated. Abbreviations: Pro-T, progenitor-T.

UCB is an enriched source of HSCs (generally defined as CD34^+^), that can be differentiated to human lymphoid cells, including T cells. However, a practical problem from a clinical utility perspective is the limited total number of HSCs that can be derived from each UCB unit. Accordingly, we investigated whether it was possible to increase the number of CD34^+^ HSCs ex vivo, using a non-xenogeneic and serum-free expansion method, without affecting cell phenotype or their capacity to differentiate. A four-step process was utilized for differentiation of HSCs to T cells (Figure 1). Firstly, freshly isolated HSCs (herein referred to as CD34^+^ HSCs) from UCB samples were expanded for five days prior to T cell differentiation (Day −5–Day 0). These were differentiated into Pro-T cells over 14 days (Day 0–Day 14) and double positive (DP) T cells after an additional 28 days of differentiation (Day 14–Day 42). CD8 single positive (SP) T cells were subsequently generated after a further seven days of activation-induced differentiation (Day 42–Day 49). Pro-T cells were broadly defined by a CD5^+^CD7^+^ phenotype, DP T cells were defined by a CD3^+/−^CD4^+^CD8^+^ phenotype and SP T cells were defined by either a CD3^+^ CD4^−^CD8^+^ (CD8^+^ SP) or CD3^+^CD4^+^CD8^−^ (CD4^+^ SP) phenotype. This process was performed with 5 independent UCB samples where cell proliferation was most rapid during HSC through to Pro-T cells, continued during development from Pro-T cells plateauing toward DP T cell development and dropped with final maturation between Day 42 to Day 49 (Figure 1). In general, for every CD34^+^ cell input, approximately 3 × 10^5^ total live cells were generated after five days of initial HSC expansion and a subsequent 49 days of differentiation (Figure 1). Of total live cells, the mean proportion of CD3^+^CD8^+^ cells was 17% at Day 49 (characterized by flow cytometric analysis), which equates to approximately 5 × 10^4^ total mature CD8^+^ T cells per HSC. This developmental progression follows the sequence normally found for thymic-based T cell differentiation [32].

**Figure 2:**
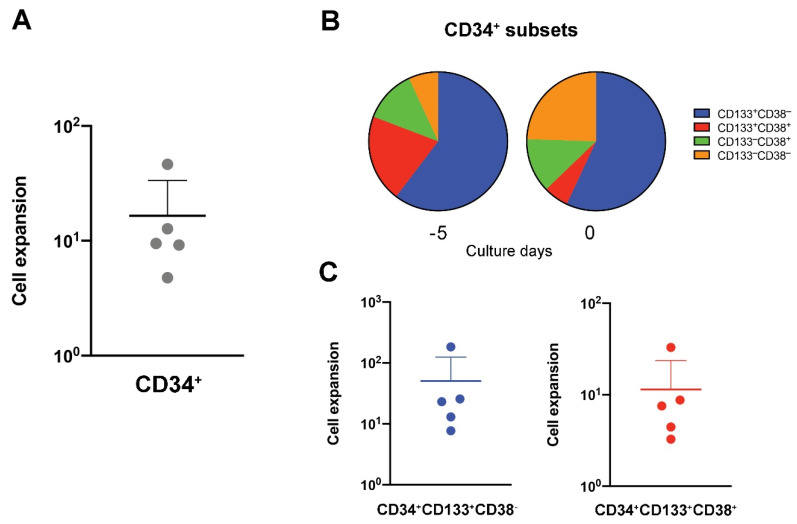
HSCs and their lymphoid progenitors are increased during expansion prior to T cell differentiation. UCB-derived CD34^+^ cells were isolated and expanded in CD34 Expansion media. (**A**) Fold change of total CD34^+^ cells, (**B**) population frequencies of CD133 and CD38 expression in the CD34^+^ population and (**C**) fold change of total CD34^+^CD133^+^CD38^−^ or CD34^+^CD133^+^CD38^+^ cells was determined after 5 days of culture. Cell number was determined using the TC20 cell counter and trypan blue staining. Individual data points represent independent biological samples; bars indicate the mean fold change ± SD. Colors represent individual cell subsets as indicated.

In vitro expansion of UCB HSCs after 5 days of culture in CD34 Expansion media, yielded a 10-fold increase in total live cells (Figure 1, CD34^+^ expansion step) with a 16-fold increase of total CD34^+^ cells (Figure 2A). The culture conditions favored CD34^+^ cell growth over any residual non-CD34^+^ cells that were present in the initial UCB samples. The CD34^+^ population can be further classified into progenitor subsets based on CD38 and CD133 expression. The majority of primitive progenitors, usually classified as CD38^low/−^ cells, are found in the CD133^+^ fraction [33,34]. Furthermore, lymphoid-primed multipotent progenitors are enriched in the CD34^+^CD133^+^CD38^−^CD45A^+^ fraction and are known to retain long-term lymphoid capacity [34]. Our CD34^+^ HSCs, with a phenotypic profile of CD133^+^CD38^−^, remained at similar percentages (>50%) to those observed in HSCs at the time of thawing through five days of expansion, suggesting that expansion does not affect the phenotypic frequency of cells with long-term lymphoid potential (Figure 2B). Additionally, we showed an average 50-fold increase in the final number of CD133^+^CD38^−^ cells after HSC expansion (Figure 2C).

CD133^+^CD38^+^ cells decreased and CD133^–^CD38^–^ increased proportionally over the five days (Figure 2B), with a 11.4-fold increase in the final number of CD133^+^CD38^+^ cells (Figure 2C). This phenotype may have the potential to form granulocyte/monocyte progenitor cells as they are enriched in the CD34^+^CD133^+^CD38^+^CD45RA^+^ fraction [34]. However, there is no clear evidence that suggests these cells lack T cell differentiation potential.

**Figure 3:**
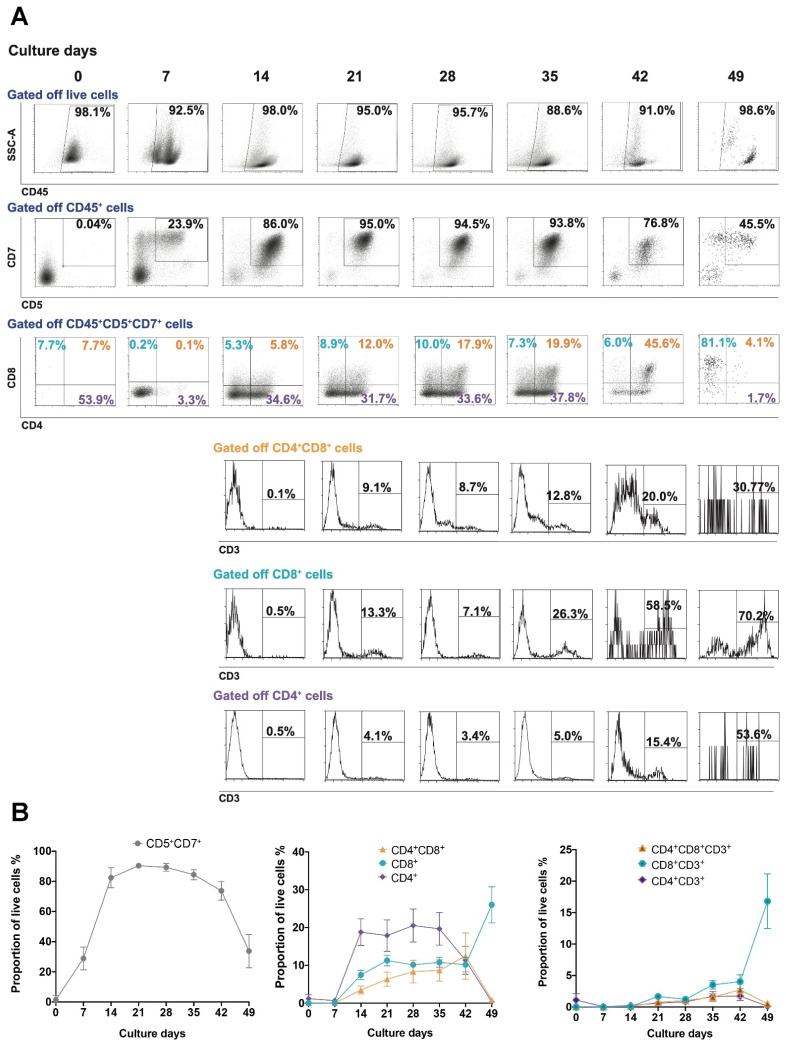
HSC-derived T cell phenotype development resembles endogenous T cell phenotype development. (**A**) Pro-T cells were induced from CD34^+^ HSCs over 14 days (Day 0–Day 14), Pro-T cells to DP T cells after an additional 28 days of culture (Day 14–Day 42) and DP T cells to SP T cells after a further 7 days of culture in mature 6F Media with anti-CD3/CD28 bead stimulation for the first 3–4 days of this last 7-day culture period (Day 42–Day 49). Firstly, all CD45^+^ cells were gated from live cells and subsequent T cell markers were analyzed. Early differentiation markers were assessed by gating CD45^+^ cells and analyzing for CD5 and CD7 expression. Late differentiation markers were assessed by gating on CD45^+^CD5^+^CD7^+^ (Pro-T) cells and analyzing for CD8^+^ and CD4^+^ expression. These cells were further analyzed for CD3 expression (no CD3^+^ cells were detected at Days 0 and 7). Representative flow plots from one cord sample are displayed. (**B**) The % proportion of live Pro-T (CD45^+^CD5^+^CD7^+^), CD4, CD8 and DP T cells with and without CD3 expression was determined by flow cytometric analysis with gating as described above and is presented as the mean proportion of live cells ± standard error of the mean (SEM) from 5 representative UCB samples. Colors represent individual cell subsets as indicated. Abbreviations: SSC-A; side scatter area.

T cell development occurs in several stage-specific differentiation steps, with earliest progenitors defined by the expression of the early differentiation markers CD7 and CD5 and a lack of CD3, CD4 and CD8. During differentiation, CD4, CD8, and CD3 are expressed as T cells mature [32]. Studies using murine stromal support cells for inducing T cell differentiation from HSCs show the generation of Pro-T cells within approximately 14 days, followed by the expression of CD4 and CD8 at approximately 28 days after the initiation of differentiation. CD3 expression was observed from Day 42 and beyond [14,15,16,18]. In our culture system, which lacks any xenogeneic stromal support cells, we observed an overall increase in Pro-T and maturing T cells over 42 days following initiation of HSC differentiation (Figure 3A,B). Flow cytometric phenotypic analysis showed increasing levels of the early differentiation markers CD5 and CD7 up to 20 days of culture (Figure 3A,B), which were maintained to 42 days, prior to Step 3 of differentiation (Figure 3A,B). From Day 14, there was increasing expression of CD4 and CD8, which continued up to Day 42 (Figure 3A,B). The increase in CD4 expression without CD3 and CD8 is indicative of the initial development of immature single positive CD4 (ISP4) cells, which was followed by the development of DP CD4^+^CD8^+^ cells (Figure 3B). The decline in Pro-T cells (CD5^+^CD7^+^) from Day 42 was associated with an increase in CD8 SP T cells, approximately ~70% of which acquired CD3 expression by Day 49 (Figure 3A). Whilst CD4 and CD8 were upregulated during development, only CD8^+^ cells co-expressing CD3 were present after the final stage of differentiation (Figure 3B). 

To mimic thymus-based positive selection, the effect of T cell receptor (TCR) and cytokine stimulation on the DP T cells was assessed. After 42 days of culture the cells were transferred to 6F Media with anti-CD3/CD28 bead stimulation for the first 3–4 days of a 7-day culture period. Beads were removed for the following 3–4 days of this 7-day period. By Day 49, CD8^+^ T cells increased while CD4^+^ T cells proportionally declined, particularly on CD3^+^ cells (CD8^+^ SP vs. CD4^+^ SP). A corresponding decline in the proportion of CD5^+^CD7^+^ Pro-T cells was also observed between Day 42 and 49 (Figure 3B), perhaps resembling the sensitivity of immature thymus cells to TCR activation during thymic-negative selection. If left in Mature media without additional cytokines or anti-CD3/CD28 bead stimulation, T cell subset proportions remained similar to those at Day 42 (data not shown). This shows that the cytokines and bead-mediated activation were responsible for driving this phenotypic development rather than a spontaneous effect of time in culture. However, despite these trends, there appeared to be donor variability in the differentiation potential of the UCB samples. Furthermore, one sample (Sample 4, Supplementary Figure S1) evidently lacked T cell development beyond the Pro-T cell stage (>80% CD45^+^CD5^+^CD7^+^ expression was observed at Day 28), however these did develop into CD8^+^CD4^−^ from Day 28 to Day 49, but apparently lacked CD3 expression. It is unclear why this occurred, but may indicate a propensity toward the NK cell lineage, given the successful development of CD5^+^CD7^+^ expression. Indeed CD56^+^CD3^−^ cells (Sample 4: ~82%) were present in this culture after the 49 days of culture (data not shown).

### 3.2. Maturation State of T Cells Differentiated from HSCs In Vitro

**Figure 4:**
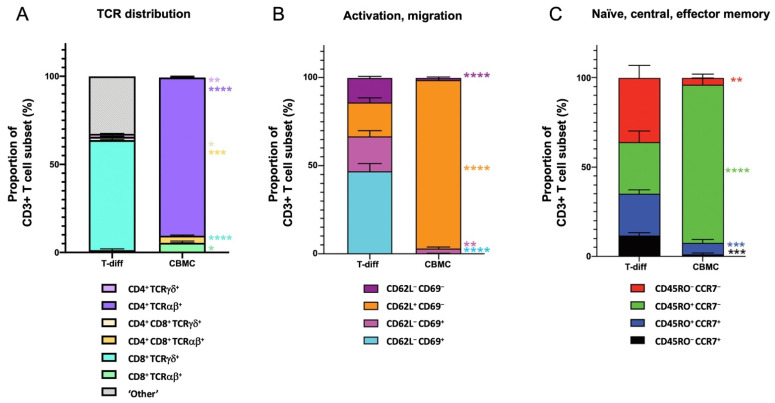
HSC-derived T cells express unique CD3^+^ profile subsets. Phenotypic profiles of T cells differentiated from UCB-derived HSCs (T-diff) were compared to naturally occurring, thymic-derived T cells directly obtained from UCBs. (**A**) Represents the distribution of TCRαβ and TCRγδ expressed on CD4^+^, CD8^+^, and CD4^+^CD8^+^ T cells (all CD3^+^). CD3^+^ cells which did not express a complete repertoire of T cell markers were classed as ‘Other’. (**B**) Represents the activation state of CD3^+^ T cells characterized by expression of CD69 and migratory potential by CD62L. (**C**) Characterizes the proportion of CD3^+^ T cells that share markers indicative of naive, central memory and effector memory phenotypes; Naive T cells were defined by CD45RO^−^CCR7^+^, effector T cells by CD45RO^−^CCR7^−^, effector memory T cells by CD45RO^+^CCR7^−^ and central memory T cells by CD45RO^+^CCR7^+^. Dead cells, doublets and debris were excluded by flow cytometric analysis, prior to gating viable, CD7^+^CD3^+^ cells into each T cell subset. Bars indicate mean % of CD3^+^ subset ± SEM of at least 4 independent biological samples, where significant differences between the proportion of T cell subsets in T-diff samples are compared to CBMC T cells * *p* ≤ 0.05, ** *p* ≤ 0.01 *** *p* ≤ 0.001 **** *p* ≤ 0.0001. Colors represent individual cell subsets as indicated. Abbreviations: CBMC, cord blood mononuclear cells; CCR7, C-C chemokine receptor type 7; TCR, T cell receptor; T-diff, T cells differentiated from UCB-derived HSCs.

HSC-derived T cells were further examined for their level of maturation and compared to T cells isolated from CBMCs. Importantly, our HSC-derived CD3^+^ T cells successfully developed expression of TCRs, with a very strong propensity toward CD8^+^ TCRγδ cells (~62% of CD3^+^ cells, Figure 4A). The co-expression of CD3 with TCRγδ indicate that the culture conditions utilized are conducive to normal TCR formation.

With respect to T cell subsets, conversely CBMC T cells were predominately CD4^+^ TCRαβ cells (Figure 4A). The higher proportion of TCRγδ T cells observed via in vitro differentiation may be due to the absence of thymic cortical epithelial cells, which are required for positive selection of TCRαβ [35]. The differentiation process also yielded CD3^+^ cells which were considered transient or incomplete CD3^+^ cells. These cells co-expressed to variable extents CD4^+^, CD8^+^, TCRαβ, and/or TCRγδ, which did not fall within typical CD3^+^ T cell subset profiles and have for these purposes been termed ‘Other’ CD3^+^ cells (Figure 4A). It is possible these cells may share some NK-T characteristics, but without yet expressing TCRs they are therefore not considered NK-T cells either. 

To further characterize the types of T cell subsets generated after differentiation, phenotypic assessment was carried out on CD3^+^ T cells. CD45RO and CCR7 expression describe phenotypic and functional subsets of T cells [36]. These subsets can also be defined by the expression of functional molecules such as CD62L, required for migration and CD69 which is linked to activation and proliferation [36]. The HSC-derived T cells were ~70% CD69^+^ (Figure 4B), supporting that in vitro differentiation culture conditions favor T cell output in an activated cell state. CBMC T cells were >90% effector memory cells (CD3^+^CD45RO^+^CCR7^−^) (Figure 4C) and CD62L^+^ but <1% CD69^+^ (Figure 4B). Conversely, T cells generated in the in vitro culture system displayed greater heterogeneity with ~10% naive T cells (CD45RO^−^CCR7^+^), ~25% effector T cells (CD3^+^CD45RO^−^CCR7^−^), ~36% effector-memory T cells (CD3^+^CD45RO^+^CCR7^−^) and ~28% central memory T cells (CD3^+^CD45RO^+^CCR7^+^). This heterogeneity would favor more diverse clinical applications. Overall, our HSC-derived T cells displayed the typical markers for thymic-derived T cells (CD45^+^CD5^+^CD7^+^CD8^+^CD3^+^TCRγδ^+^) and co-expressed typical naive/central memory markers demonstrating extensive T cell development.

### 3.3. Cytotoxic Function of T Cells Differentiated from HSCs In Vitro

**Figure 5:**
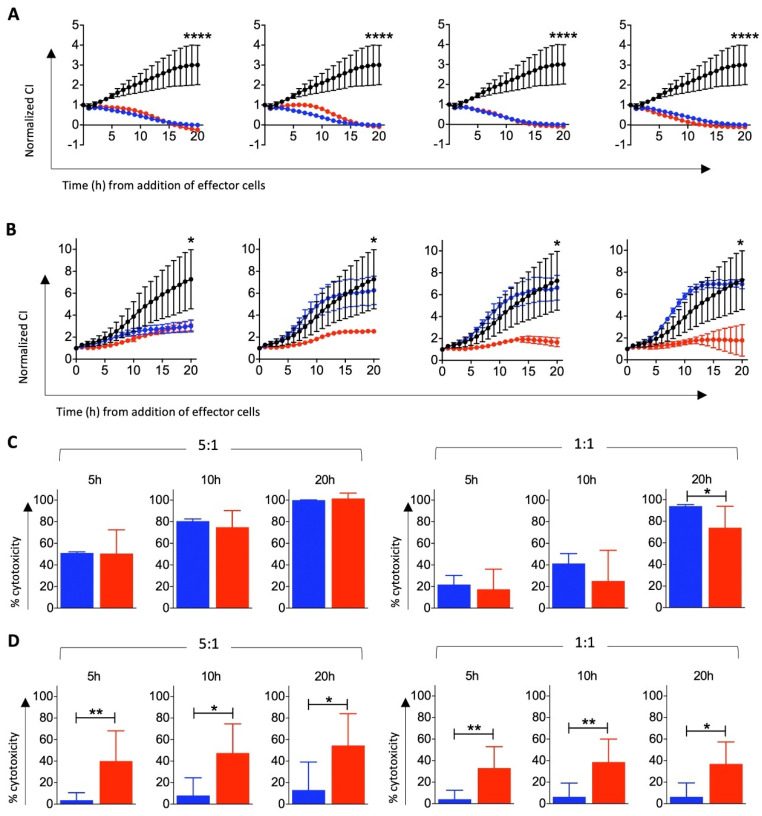
HSC-derived T cells induce killing of ovarian cancer cells in vitro. T cells were generated from HSCs for 42 days and transferred to 6F media in the presence of anti-CD3/CD28 DynaBeads^®^ for the first 3–4 days of a 7-day culture, to induce polyclonal T cell activation. (**A**) OVCAR-3 and (**B**) MES-OV target cells were co-cultured with the HSC-derived T cells (red) or T cells isolated from CBMCs (blue) at an effector to target (E:T) ratio of 5:1. Target cell alone controls (black) were maintained in parallel. Their cytotoxicity response was monitored in real time using xCELLigence, where a decrease in Normalized Cell Index (CI) is indicative of target cell death relative to target cells alone. Each plot is representative of a single donor performed in technical triplicate. Efficiency of (**C**) OVCAR-3 and (**D**) MES-OV target cell killing was quantitated at 5 h, 10 h and 20 h and presented as average % cytotoxicity ± SD pooled from 4–8 biological replicates. * *p* ≤ 0.05, ** *p* ≤ 0.01, **** *p* ≤ 0.0001. Abbreviations: CI, Cell Index; h, hour.

To determine whether HSC-derived T cells could induce tumor cell killing, cultures were harvested at Day 49 (after activation with 6F Media and anti-CD3/CD28 beads, as described above) and cytotoxic activity was assessed in vitro. T cells isolated from four donor matched CBMCs were maintained in T cell expansion media and assessed in parallel as a positive control for cytotoxic capacity. All effector cells were tested against the ovarian cancer cell lines OVCAR-3 and MES-OV (Figure 5). Whilst not all live cells from HSC-differentiated cultures displayed hallmark T cell phenotypes (Figure 4), the cytotoxic effect in Figure 5 is understood to be driven by the presence of the T cells produced as a result of the differentiation process. 

HSC-derived T cells from each donor assessed were highly cytotoxic against OVCAR-3 cells as shown by a significant reduction in Normalized CI over 20 h (Figure 5). Cytotoxic function of these effector cells was comparable to CBMC T cells (Figure 5A). Greater donor-variation was observed in MES-OV co-cultures (Figure 5B). Cytostatic and cytotoxic responses were observed when HSC-derived T effector cells were used. In contrast, no cytotoxic responses and only one of four CBMC T cell donor elicited a cytostatic response in MES-OV co-cultures suggesting enhanced functional capacity of the T cells differentiated from HSCs.

This is further supported by the direct comparison of pooled % cytotoxicity of OVCAR-3 (Figure 5C) and MES-OV (Figure 5D) co-cultures at both 5:1 and 1:1 E:T ratios. T cells derived from HSCs are significantly more effective at eliminating MES-OV cells in vitro. The underlying reasons for these differences are currently unclear.

## 4. Discussion

Given their central role in cancer therapy and defense against opportunistic infections, clinically relevant strategies are needed for the generation of large numbers of T cells. This is particularly true for cancer patients where the immune system is often severely compromised from chemotherapy. In addition, the advent of CAR-T cell technology has been successful for autologous treatment of blood cancers, but the process is expensive, time consuming and limited by the number of patient T cells which can be harvested. These deficiencies have stimulated great interest in ‘off-the-shelf’ allogeneic cellular immunotherapies. In vitro directed T cell differentiation from HSCs offers a logical strategy to generate large numbers of exogenous killer cells, with the potential to reduce cost and provide ‘off-the-shelf’ T cell therapy. One readily available source is UCB HSC. In this study we used a molecularly defined T cell induction system, free of xenogeneic serum and stroma cells, in which 1x UCB HSC gave rise to 5 × 10^4^ T cells in 49 days of differentiation. Multiple cell subtypes were developed under different stimulation conditions, with CD8^+^ T cells (γδ) preferentially produced. There was, however, variability observed between UCB donors which affected differentiation efficiency, phenotype distribution, and the number of T cells generated.

Human T cells have been previously generated in vitro [15,37,38,39,40], however, these approaches have largely relied on using mouse-derived OP9 stromal cell lines that ectopically express the Notch ligand Delta-like-1 (DLL-1) or Delta-like-4 (DLL-4) (OP9-DL) [18,41]. The OP9-DL system is efficient at inducing commitment to the T cell lineage, sequentially generating CD4^−^CD8^−^ double negative, ISP4 and DP T cells but low levels of CD3 and TCR expression and hence inefficient production of mature SP4 and SP8 T cells [14]. The OP9 system is also highly variable and thought to be due to loss of differentiation inducing molecules [42]. Embryoid bodies (EBs) in conjunction with the OP9-DLL-4 system, have allowed iPSCs or embryonic stem cells (ESCs) to be directed towards HSC-like cells capable of T cell differentiation. The CD34^+^ cells from EBs produced ISP4 and DP T cells with visible CD3 expression, but the production of conventional mature T cells (SP8 and SP4) was again limited [15,16]. Furthermore, the common use of xenogeneic serum-containing medium and xenogeneic stromal cells in these models also limits their translation to the clinic. 

Notch signaling is vital for inducing T cell differentiation from HSCs [43]. Reimann et al. utilized immobilized human DLL-4–Fc to produce Pro-T cells from UCB [44]. This approach was stromal cell-free, however FBS was used, again limiting its adaptability. To address this, Shukla et al. established a defined in vitro niche, combining DLL-4–Fc and vascular adhesion molecule-1 with cytokine supplementation. CD7^+^ Pro-T cells derived from this system showed thymus-seeding potential and the reconstitution of the peripheral T cell compartment in immunodeficient mouse recipients [45]. The ability to obtain mature functional human T cells in long-term cultures, however, has remained elusive. In overcoming this barrier, one study has found that the inclusion of ascorbic acid in immobilized DLL-4–Fc cultures made it possible to develop CD4^+^CD8^+^ DP and TCRαβ^+^CD3^+^ SP T cells [46]. More recently, artificial thymic organoids, based on the mouse MS5 cell line which expresses human DLL-1 or DLL-4, induced T cell differentiation from HSC, ESC, and iPSC, similar to that of the human thymus. They generated ISP4 and DP cells and in particular they showed efficient positive selection [47,48]. By week 5, 90% of the cells were CD3^+^TCRαβ^+^ and approximately 80% of these cells were functional CD8 SP cells [48,49]. However, the dependence on the mouse stromal cell lines precludes their clinical translation and there is also the issue of CD3^+^TCRαβ^+^ T cells needing to be purged of graft-versus-host alloreactivity.

The development of a highly efficient support cell-free culture system that generates mature T cells as described in the present study, is more likely to have an immediate translational impact [50]. The initial step in the process was a five-day expansion of UCB-derived HSC. While inducing a 16.5-fold expansion, the culture conditions retained the CD34^+^CD133^+^CD38^−^CD45A^+^ HSC subset enriched for long-term lymphoid potential [34]. From each cord sample, approximately 5 × 10^6^ CD34^+^ HSCs were isolated. As each individual CD34^+^ HSC generates ~5 × 10^4^ mature CD8^+^ T cells using the differentiation method described here, each cord sample has the potential to create approximately 2.5 × 10^11^ T cells (through differentiation of all CD34^+^ cells). This is orders of magnitude higher than typical autologous T cell manufacture systems [51]. The T cell differentiation progressed through the CD5^+^CD7^+^ Pro-T cell stage to immature DP T cells by 42 days. Given that CD8^+^ T cells are effective killers of malignant cells and are often used in CAR-based immunotherapies to enhance tumor eradication [52], a key hurdle for the successful in vitro development of cytotoxic T cells is the progression of CD3^+/−^CD4^+^CD8^+^ immature T cells through to TCR^+^CD3^hi^CD8^+^CD4^−^ cells. In the thymus, this sequential molecular rearrangement is induced by positive selection which occurs by binding of the CD3/TCR with its cognate major histocompatibility complex (MHC) Class I or II/peptide complex presented by cortical epithelial cells [35]. MHC Class I engagement induces the downregulation of CD4 and Class II the downregulation of CD8 [53]. We attempted to drive this by culturing the CD3^+/−^CD4^+^CD8^+^ immature T cells via cytokine co-stimulation [30] and with anti-CD3/CD28 coated beads. The most obvious effect was the directed induction of CD8^+^ TCRγδ T cells. Since positive selection of CD4^+^ cells require co-engagement of the TCR with MHC class II ideally presented on thymic epithelium, [54], it is unsurprising that CD4^+^ cells were not induced herein because the MHC Class II selecting ligands were not present. As the in vitro differentiation process involves predominately cells that only express MHC class I, this would explain the development toward mature CD8^+^ T cells. 

For prospective immunotherapeutic applications, TCRγδ cells have some advantages: their restricted TCR repertoire and lack of recognition of MHC/peptide complexes, precludes their propensity to induce GVHD in the allogeneic setting. Nor are they likely to cause autoimmunity. In fact, they can ameliorate this disease via release of immunoregulatory cytokines [55,56]. TCRγδ T cells typically do not react against normal healthy cells and do not follow equivalent negative selection screening as TCRαβ T cells. Instead, they recognize stress related molecules including non-protein phosphoantigens, isoprenoid pyrophosphates, alkylamines, non-classical MHC class I molecules MICA and MICB, as well as heat shock-derived peptides on target cells without requiring antigen processing and MHC presentation [56]. Accordingly, it is likely the differentiated TCRγδ T cells created here will favor recognition of “abnormal” cells, such as those in infections and particularly cancer cells rather than normal healthy cells. This remains to be verified for clinical translation. 

One area that needs attention in this system is the presence of cells designated as ‘Other’ (Figure 4A), which expressed CD3^+^ but not typical TCRαβ or TCRγδ co-expression with CD4 and CD8 subsets. It is unknown if these cells may pose any potential safety risks. To address this, the cells termed ‘Other’ could be removed by the positive selection of CD3^+^ TCRγδ^+^ cells by fluorescence-activated cell sorting or isolation with antibody-coated beads before the product could be adopted clinically.

On the other hand, TCRαβ T cells can cause both GVHD and autoimmunity. From a safety perspective, TCRαβ T cells generated in vitro for allogeneic therapy would need to be subjected to recipient specific, tolerance inducing negative selection, e.g., by dendritic cells [35,57]. Their broader TCR repertoire also predisposes them to causing autoimmune disease. Both of these health risks could be addressed by replacing the TCRαβ with a CAR [58,59], but these cells would then lack the advantages of a TCR specificity repertoire.

The presence of elevated CD69 expression in these in vitro differentiation conditions, indicated the in vitro HSC-derived T cells present an activated phenotype, geared toward proliferation and function. Most importantly, as a result of this combination of activation factors, these cells were highly cytotoxic to the ovarian cancer cell lines OVCAR-3 and MES-OV. In comparison, T cells derived from UCB were similarly cytotoxic to OVCAR-3 but had no effect on the MES-OV cells. The precise mechanism of action of this polyclonal activated killing is unknown, but if the effector cells were “rested” by culture for a further three days in medium without stimulants, the killing efficacy was lost (data not shown). Regardless, this present study clearly shows the induced T cells have potent cytolytic function and represent a therapeutic vehicle for allogeneic CAR-T cells, being TCRγδ^+^. An interesting follow up study could be to further profile the subtypes of TCRγδ in these cells given that Vγ9Vδ2 T cells are promising candidates for cellular tumor immunotherapy [60].

These cells are anticipated to lack GVHR in the allogeneic setting [13,57]. Furthermore, the heterogeneity in the type of T cells produced from HSCs may have clinical benefit given the diversity of immune responses that could synergize for cancer destruction. Nevertheless, further preclinical studies are warranted prior to their use as CAR-T therapies, including the introduction of cancer specificity through CAR incorporation and antigen specific tumor eradication assessment.

In summary, this culture system serves as a stand-alone, simple, support-cell free manufacturing technique for inducing CD8^+^ cytolytic T cells. There is application potential to enable immune reconstitution for a variety of diseases and provides an important piece of the puzzle for unlocking ‘off-the-shelf’, affordable, T cell-based cancer immunotherapy.

## Data Availability

The data presented in this study are available on request from the corresponding author. The data are not publicly available due to Cartherics Pty Ltd. confidentiality.

## Supplementary Materials

The following are available online at https://www.mdpi.com/article/10.3390/cells10102631/s1, Table S1: Monoclonal antibodies used for phenotypic analysis of cell subsets. Figure S1: HSC-derived T cells incrementally express T cell markers over 49 days of differentiation and display cord-to-cord variability.
