# Supplementary material for: ‘Off-the-Shelf’ Immunotherapy: Manufacture of CD8+ T Cells Derived from Hematopoietic Stem Cells
Source: Cells. 2021 Oct 2;10(10):2631. doi: 10.3390/cells10102631 (PMC8534391; doi:10.3390/cells10102631)
Supplement: Supplementary file 1 [file cells-10-02631-s001.zip › cells-1331388-supplementary.pdf]

cells MDPI

Supplementary Materials

Table S1. Monoclonal antibodies used for phenotypic analysis of cell subsets. Abbreviations: APC, allophycocyanin; CCR7, C-C chemokine receptor type 7; FITC, fluorescein isothiocyanate; IgG, immunoglobulin G, PE, phycoerythrin; TCR, T cell receptor.

Antigen	Fluorophores	Clone & Isotype
CD3	VioGreen™	REA613 & REA control (S)
	FITC	REA613 & REA control (S)
	PE	REA613 & REA control (S)
CD4	APC	REA623 & REA control (S)
	VioBlue™	REA623 & REA control (S)
	PE-Vio®770	REA623 & REA control (S)
	VioBright™ FITC	REA623 & REA control (S)
CD5	PE	REA782 & REA control (S)
	APC	REA782 & REA control (S)
CD7	PE-Vio®770	CD7-6B7 & mouse IgG2aκ
	APC-Vio®770	CD7-6B7 & mouse IgG2aκ
	APC	CD7-6B7 & mouse IgG2aκ
CD8	VioBlue™	REA734 & REA control (S)
CD34	APC	AC136 & mouse IgG2aκ
	VioBlue™	AC136 & mouse IgG2aκ
	PE-Vio®770	AC136 & mouse IgG2aκ
CD38	VioBlue™	REA671 & REA control (S)
	VioBright™ FITC	REA671 & REA control (S)
CD43	PE	DF-T1 & mouse IgG1κ
CD45	VioGreen™	REA747 & REA control (S)
	APC-Vio®770	
CD45RO	FITC	REA611 & REA control (S)
CD56	PE-Vio®770	REA196 & REA control (S)
	FITC	REA196 & REA control (S)
	APC	REA196 & REA control (S)
CD62L	PE	REA615 & REA control (S)
CD69	APC-Vio®770	REA824 & REA control (S)
CD133/1	PE-Vio®770	REA753 & REA control (S)
CD197 (CCR7)	PE-Vio®770	REA546 & REA control (S)
	VioBlue™	REA546 & REA control (S)
TCRαβ	APC-Vio®770	REA652 & REA control (S)

TCRγδ	PE	REA591 & REA control (S)

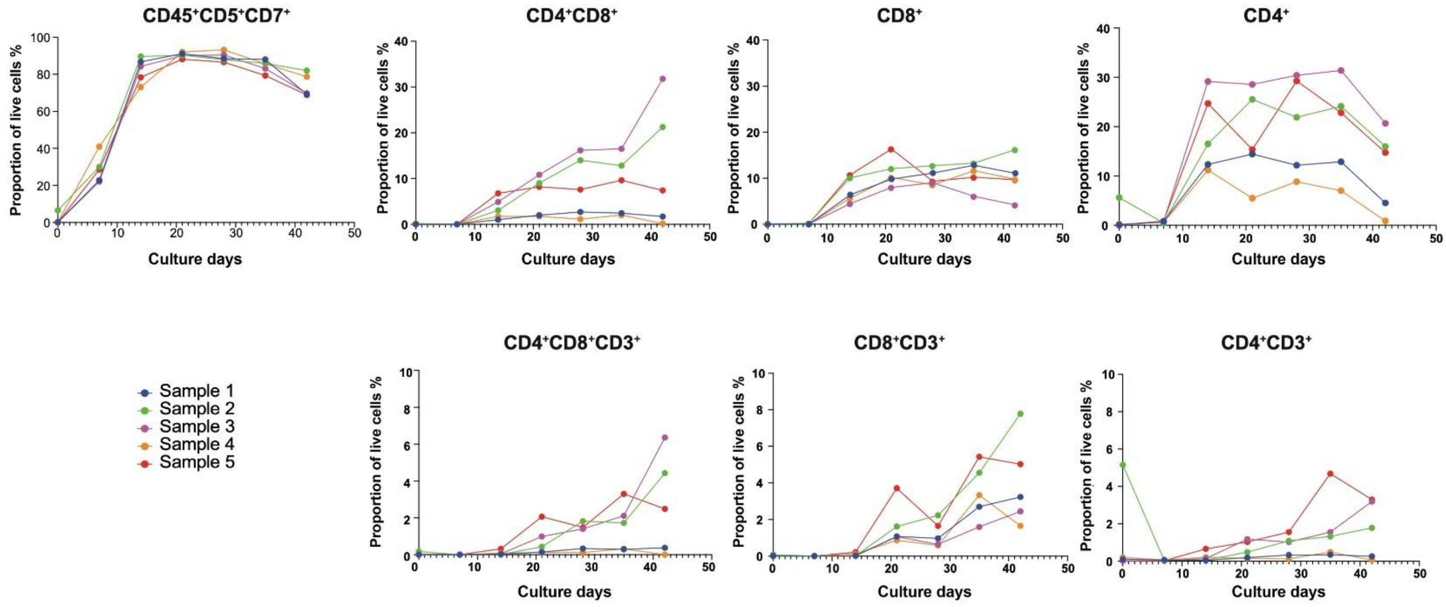

Figure S1. HSC-derived T cells incrementally express T cell markers over 49 days of differentiation and display cord-to-cord variability. The % positive cell frequencies of Pro-T, CD4, CD8 and DP T cells with and without CD3 expression are displayed as a proportion of total live cells; representative of 5 UCB samples. CD45⁺ cells were gated from live cells and subsequent T cell markers were analyzed. CD45⁺ cells were analyzed for CD5⁺CD7⁺ co-expression; CD5⁺CD7⁺ cells were assessed for CD4⁺ and/or CD8⁺ expression, then each CD4⁺, CD8⁺ and DP subset was further analyzed for CD3 expression. Similar development trends were observed with the exception of one (Sample 4) which didn't progress beyond Pro-T cells; it did however, produce NK cells (data not shown). Colors represent individual UCB samples as indicated.
